# International Congress on Tuberculosis

**Published:** 1908-03

**Authors:** 


### International Congress on Tuberculosis.

The JOURNAL is requested to publish the following:

SAN ANTONIO, TEXAS, January 30, 1908.

*Dr. .................., President............County Medical Society.*

DEAR DOCTOR: The International Congress on Tuberculosis meets in Washington next September. This great educational organization comes to this country for the first time on the invitation of the National Association for the Prevention of Tuberculosis, which is a great working sanitary institution. It is impossible to overestimate the importance of this world congress of philanthropic sanitarians. It marks an epoch in sanitation and must necessarily lead to such widespread interest in the prevention of tuberculosis that much good must result.

The President of the United States and the Governors of more than thirty States have become actively interested and boards of health and medical associations everywhere are giving enthusiastic encouragement. We have the hearty co-operation of our Governor and State Health Officer.

Texas with her intelligent citizenship and her special needs should not take a second place in this important movement, but should stand first in promoting every enterprise looking to the public health, especially concerning tuberculosis.

The National Association has appointed a Texas committee to make some organized efforts, and this committee has urgently requested the Board of Trustees of the State Medical Association to interest the county societies, the great pivot upon which so much depends. At a recent meeting of the board the chairman and secretary were authorized to present this matter to your society, and we would respectfully suggest:

First. That those who can, join the International Congress on Tuberculosis. The cost is $5, which will include the published transactions of the coming meeting in Washington. Dr. W. S. Carter, President of the Texas committee, or Dr. M. M. Smith, Secretary, will be pleased to receive applications.

Second. That you influence other public-spirited citizens to become members.

Third. That you enlist the interest of the city and county authorities, boards of health, school boards, women's clubs, etc.

Fourth. That, if practicable, you send an accredited delegate to the Congress, such delegate to be a member.

We leave the matter to the wisdom of your body, and enclose a postal card hoping to hear what action you have taken.

Yours very truly,

J. S. Lankford, M. D., President,
San Antonio, Texas.

W. R. Thompson, M. D., Secretary,
Fort Worth, Texas.

For the Board of Trustees, Texas State Medical Association.

